# Effects of Trace Elements and Vitamins on the Synthesis of Steroid Hormones in Follicular Granulosa Cells of Yak

**DOI:** 10.3390/vetsci11120619

**Published:** 2024-12-03

**Authors:** Yanbing Lou, Tingting Yang, Yanqiu Zhu, Chenglong Xia, Hengmin Cui, Huidan Deng, Yixin Huang, Jing Fang, Zhicai Zuo, Hongrui Guo

**Affiliations:** 1 College of Veterinary Medicine, Sichuan Agricultural University, Wenjiang, Chengdu 611130, China; louyanbing@stu.sicau.edu.cn (Y.L.); yangtingting@stu.sicau.edu.cn (T.Y.); zhuyanqiu@sicau.edu.cn (Y.Z.); xiachenglong@stu.sicau.edu.cn (C.X.); chm2020@sicau.edu.cn (H.C.); denghuidan@sicau.edu.cn (H.D.); yxhuang@sicau.edu.cn (Y.H.); fangjing4109@163.com (J.F.); 2 Key Laboratory of Animal Diseases and Environmental Hazards of Sichuan Province, Sichuan Agriculture University, Wenjiang, Chengdu 611130, China

**Keywords:** granulosa cells, steroid hormone, trace elements, vitamins, yaks

## Abstract

Yak (*Bos grunniens*), a special breed of cattle on the Qinghai–Tibet Plateau, has low fertility due to nutritional deficiency, especially the trace elements. The steroid hormones estradiol (E2) and progesterone (P4) synthesized by yak follicular granulosa cells (BGCs) are involved in the entire reproductive process. In the present study, we investigated the effects of trace elements and vitamins on yak follicular GCs, including the cellular activity, the synthesis of E2 and P4, and the expression of genes related to steroid hormone synthesis. The results showed that moderate supplementation of vitamin D3 (VD3), strontium (Sr), manganese (Mn), and selenium (Se) enhanced granulosa cell activity. Within the safe dose range, the addition of vitamin A (VA), VD3, cobalt (Co), Sr, copper (Cu), Mn, Se, and chromium (Cr) significantly increased the synthesis of E2 by GCs, while the addition of VA, vitamin C (VC), VE, zinc (Zn), Sr, Cu, and Cr enhanced the production of P4 in GCs. The changes in steroid synthesizing genes were consistent with the changes in hormone synthesis. This study provides an experimental basis for the addition of trace elements to improve the production performance of yaks.

## Simple Summary

Yak is a unique species of highland cattle that inhabits cold regions year-round. Poor soil conditions lead to low nutrient content in forage, making yaks susceptible to trace element deficiencies. This deficiency results in reduced reproductive rates and overall productivity in yaks. Hormones synthesized by the granulosa cells of yak follicles play a crucial role in yak reproduction. In this study, the changes in cell viability, hormone secretion, and hormone synthesis-related genes were observed by adding metal trace elements and vitamins to the culture medium of cultured granulosa cells of yak follicles in vitro. The results showed that the addition of certain trace elements and vitamins had a positive effect on the activity and hormone secretion of granulosa cells in yak follicles. These findings offer a potential solution for enhancing the reproductive performance of female yaks affected by trace element deficiencies.

## 1. Introduction

Yak (*Bos grunniens*) is a key domestic animal that is distributed on the Qinghai–Tibetan Plateau and nearby alpine or subalpine regions [1]. Yak can provide a variety of important means of subsistence and production, such as milk, meat, hides, and dung. Yaks are semi-domesticated grazing animals. They are generally not provided with prepared fodder [2]. Yaks generally live in a harsh environment and are usually kept at altitudes of 3000–5400 m. During the cold season, prolonged snowfall causes a shortage of pasture, and the high altitude results in soil micronutrient deficiencies, which lead to yak malnutrition and growth retardation [3].

Yaks are usually conceived in late summer and give birth in early summer. Female yaks have poor sexual maturity and low fertility, with an average annual reproduction rate of less than 60 per cent. Studies have shown that female yaks may ovulate as low as 4–5 times per year [4]. Yaks have long calving intervals and unclear estrus periods, and even highly productive yaks can only calve 4–5 times in their lifetime [5]. There are many reasons that affect low fertility in cattle, such as heat stress, lipid metabolism, micronutrient deficiencies, etc. [6,7]. In addition to their role as nutritional support for the health and survival of the organism, micronutrients can also act as catalysts for the synthesis of certain organic substances. Trace elements are closely related to reproduction, mainly by affecting hormone secretion in the endocrine system, enzyme activity, and the organization of reproductive organs, thus affecting reproduction. Some studies have shown that injections of trace elements and vitamins increase a cow’s overall chance of becoming pregnant [8]. Micronutrient and vitamin supplementation reduces the incidence of mastitis and retained placenta in dairy cows [9]. Mineral trace element supplementation also increases yak milk yield and milk protein lactoferrin concentration [10]. Micronutrient deficiencies or exposure to toxic micronutrients greatly increase the risk of pregnancy failure [11]. In an in vitro study, adding zinc sulfate to the culture medium of yak oocytes increased the glutathione content and mRNA expression levels of antioxidant enzymes, decreased ROS levels, and improved oocyte maturation and the in vitro fertilization rate [12]. The trace element selenium had a similar effect. Adding sodium selenite to cultured yak oocytes in vitro could increase the activity of glutathione peroxidase (GSH-Px) and the rate of embryo blastocyst [13].

GCs in the follicle are essential for oocyte growth and development and ovulation. GCs mainly provide nutritional support to oocytes, and more importantly, GCs regulate oocyte development through the secretion of steroid hormones, and it has been shown that there is a close relationship between ovarian ovulation and apoptosis in GCs [14,15,16]. Steroid hormones include progesterone (P4), estradiol (E2), etc., and these hormones have a key role in maintaining the reproductive physiology of female animals [17]. Granulosa cells have been shown to play a pivotal role in follicular steroidogenesis and are also important in maintaining the follicular microenvironment and regulating oocyte growth and development [18]. The effect of trace elements on GCs has gradually become a hot research topic in recent years. Some studies have shown that pathways and genes related to iron death caused by iron excess may be involved in regulating the proliferation and function of GCs [19]. Trace element Se protects follicular granule cells from mercury-induced apoptosis by inhibiting the ATF6/CHOP pathway in chickens [20]. It has been shown that the trace element Zn protects against premature ovarian failure in rats by reducing granulosa cell apoptosis [21]. V_E_ and Se supplementation reduces hydrogen peroxide damage to bovine granulosa cells [22]. However, excessive trace elements can also adversely affect the development and function of follicle granulosa cells. Copper exposure induces steroidogenesis disorders through the FSHR/CYP19A1 pathway [23]. Fe^2+^ can directly inhibit granulosa cell proliferation by regulating the ROS-mediated p38MAPK/p53/p21 pathway [24]. Cr induces apoptosis of granulosa cells through the p53-SIRT1 network [25]. Supplementation of vitamin D3 to the culture medium of follicular granulosa cells in vitro promoted steroid hormone secretion and steroidogenic enzyme mRNA expression in granulosa cells [26]. Vitamin C can mitigate the negative effects of heat stress on reproductive processes by regulating amino acid metabolism in granulosa cells [27]. The present study shows that the effects of small amounts of vitamin supplementation on granulosa cells are beneficial, but the effects of excessive vitamin supplementation on granulosa cells are less studied.

In this experiment, we added trace elements and vitamins into the medium of primary BGCs in vitro, and detected granulosa cell activity, estradiol and progesterone secretion, and steroid hormone synthesis genes, so as to preliminarily evaluate the effects of trace elements and vitamins on follicular granulosa cell proliferation and steroid hormone secretion in yaks. The present study is of great significance for improving yak breeding efficiency and promoting yak production.

## 2. Materials and Methods

### 2.1. Materials and Reagents

Zinc chloride (ZnCl_2_), nickel chloride (NiCl_2_), manganese sulfate (MnSO_4_), cobalt chloride (CoCl_2_), strontium chloride (SrCl_2_), copper sulfate (CuSO_4_), sodium selenite (Na_2_SeO_3_), chromium trichloride (CrCl_3_) came from Chengdu Kelong Chemical Co., Ltd. (Chengdu, China). Vitamin A (cat no V830149) came from Macklin (Shanghai, China). Vitamin B12 (cat no V8040), vitamin C (cat no A8100), vitamin D3 (cat no V8070), vitamin E (cat no V8010), MTT Cell Proliferation and Cytotoxicity Assay Kit (cat no M1020) came from Solarbio (Beijing, China). Bovine Estradiol (E2) ELISA Kit instruction (cat no MM-0023O1), Bovine Progesterone (P4) ELISA Kit instruction (cat no MM-50918O1) came from Jiangsu Meimian Industrial Co., Ltd. (Yancheng, China). UNlQ-10 Column Trizol Total RNA Isolation Kit (cat no B511321) came from Sangon Biotech (Shanghai) Co., Ltd., (Shanghai, China). Hifair^®^ III 1st Strand cDNA Synthesis SuperMix for qPCR (gDNA digester plus) (cat no 11141ES60) came from Yeasen Biotechnology (Shanghai) Co., Ltd. (Shanghai, China). Testosterone (cat no T102169) came from Aladdin (Shanghai) Co., Ltd. (Shanghai, China). SYBR PRIME qPCR (FASTHS) (cat no BG0014) came from Baoguang Biotechnology (Chongqing) Co., Ltd. (Chongqing, China). Antibody FSHR (cat no A1480) came from ABclonal Technology Co., Ltd. (Wuhan, China).

### 2.2. Cell Extraction and Characterization

Yak ovaries (n = 18, ranging in age from 3 to 4 years) were obtained from a local slaughterhouse (Chengdu, China). Yak ovary tissues were placed in PBS containing penicillin–streptomycin–amphotericin B (triple antibiotic) at 37 °C and sent to the laboratory cellular compartment within 2 h for subsequent manipulation. The ovarian tissue was first placed in a sterile beaker and washed with PBS supplemented with 2-fold double antibody. A 5 mL syringe needle was used to puncture a 4–6 mm healthy follicle (healthy follicles are characterized as translucent and vascularized follicles), and the follicular fluid was aspirated and placed into a 10 mL centrifuge tube for temporary storage. After collection, the follicular fluid was blown and mixed with a pipette, and then filtered through a 200-mesh (74 mm) steel sieve to remove excess tissue and impurities; the filtrate was collected, centrifuged at 1500 rpm/min for 3 min, the supernatant was discarded, and the cells resuspended with PBS containing dual antibodies, centrifuged at 1500 rpm/min for 3 min, the supernatant was discarded, and the procedure was repeated once. The cells were centrifuged with DMEM/F12 at 1500 rpm/min for 3 min, and the supernatant was discarded. Cells were resuspended with complete medium; 5 × 10^6^ cells were counted and inoculated into T25 culture flasks and placed in a cell culture incubator at 37 °C with 5% CO_2_. The subsequent experiments were performed when the cell growth was set at 80%.

After BGCs were cultured, subcellular localization of FSHR in BGCs was performed using immunofluorescence staining. The cells were inoculated in cell culture plates with crawler plates and fixed in paraformaldehyde when the cell growth reached about 60%; then, they were closed with a sealing solution at room temperature, FSHR primary antibody was added and incubated, then FITC-labeled fluorescent secondary antibody was added and incubated at room temperature and protected from light for 1 h, the crawler plates were removed and then sealed with an anti-fluorescence quenching sealer, and then placed in a confocal laser microscope for observation and photographs.

### 2.3. Cell Culture and Treatments

Yak follicle GCs were isolated and identified and cultured in 96-well cell culture plates at 5 × 10^3^ cells per well. Amounts of 0.1% bovine serum albumin, 1% penicillin–streptomycin–amphotericin, and 10–6 M testosterone were added to DMEMF/12. The culture conditions were 37 °C and 5% CO_2_ concentration. Zinc, nickel, manganese, cobalt, strontium, copper, sodium, chromium, vitamin A, vitamin B12, vitamin C, vitamin D3, and vitamin E were weighed with an electronic analytical balance and dissolved with the DNase/RNase-Free Distilled Water, Dimethyl sulfoxide, or absolute ethanol to a concentration of 200 Mm in the mother liquor, and the cells were treated for 24 h by setting the gradient concentration treatment group, control group without micronutrient or vitamin treatment, and blank control group. According to the results of the cell viability assay, the concentration of trace elements at which the critical BGCs’ activity decreased significantly, the BGCs’ activity began to decrease significantly, the BGCs’ activity decreased to about 80%, and the BGCs’ activity decreased to about 50% were determined as the optimal treatment concentration.

### 2.4. Cell Activity Assay

After the cells were processed for 24 h, the supernatant was carefully aspirated, and the 96-well plate was washed with PBS 2–3 times, 90 μL of fresh culture medium was added, 10 μL of MTT (M1020, Solarbio, Beijing, China) solution was added, and the incubation was continued for 4 h. Then, the supernatant was aspirated off, 110 μL of Formazan dissolution solution was added to each well, and the plate was placed on a shaker for low-speed shaking for 10 min, so as to fully dissolve the crystals. The crystals were fully dissolved. The absorbance values of each well were measured at 450 nm using an enzyme-linked immunoassay reader.

### 2.5. ELISA

To normal culture cells at a density of 60–70%, different concentrations of trace elements and vitamins were added and continued to culture for 24 h; the culture medium was collected, centrifuged at 2000–3000 rpm to remove the supernatant, and the Estradiol and progesterone concentration kit (MM-0023O2, MM-50918O2, MEIMIAN, Yancheng, China) were taken out. They were returned to room temperature, the sample wells to be tested were set up; to the standard wells and blank control wells in the coated microtiter wells, the specimen, standard, and HRP-labeled detection antibody were added in turn, after warm incubation and thorough washing. In the pre-coated microtiter wells of E2 or P4 antibody, the sample wells to be tested were set up, and to the standard wells and blank control wells, the specimen, standard, and HRP-labeled detection antibody were added sequentially, after warming and thorough washing. The color was developed with the substrate TMB, which was converted to blue by catalysis of peroxidase and finally to yellow by acid. There was a positive correlation between the shade of color and the presence of E2 or P4 in the sample. The absorbance values of each well were measured at 450 nm using an enzyme-linked immunoassay reader.

### 2.6. Quantitative Reverse Transcription PCR (RT q-PCR)

Cell samples were stored in an ultra-low-temperature refrigerator at −80 °C after collection. Total RNA was extracted using the Animal Total RNA Isolation Kit (RE-03014, Foregene, Chengdu, China) according to the instructions. Then, RNA was reverse transcribed into cDNA using the PrimeScript^®^ RT Kit (RR047A, Takara, Japan) according to the instructions. Primers were designed and synthesized by Sangyo Bioengineering (Shanghai) Ltd., (Shanghai, China) (Table 1). After primer design, q-PCR reactions were performed using SYBR PRIME qPCR (FASTHS) (BG0014, BaoGuang, Chongqing, China) with the following reaction procedure: pre-denaturation at 95 °C for 3 min; denaturation at 95 °C for 10 s; annealing for 30 s for 44 cycles; 95 °C for 10 s; 72 °C for 10 s (the rate of change in temperature was 0. 5 °C/s). The reactions were detected using LightCycler^®^480 Real-Time PCR System (Bio-Rad, Hercules, CA, USA). β-actin gene was used as an internal control. The expression level of mRNA was determined by 2^−ΔΔCT^ method.

### 2.7. Statistical Analysis

The data were analyzed by GraphPad Prism 8.0 and SPSS software (version 21.0, IBM Corporation, Armonk, NY, USA). Independent samples t-tests or one-way analysis of variance (ANOVA) were used to compare data between groups. Data were represented as means + standard deviation. *p* < 0.05 indicates a significant difference.

## 3. Results

### 3.1. Identification of BGCs

**Figure 1:**
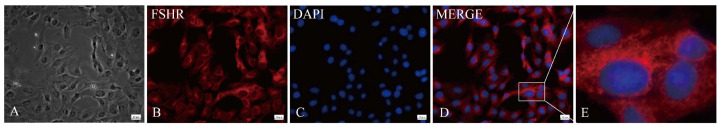
Identification of ovarian granulosa cells in yak. Bright-field image of yak follicular granulosa cell (**A**). The marker of ovarian granular cells was detected with antibodies against FSHR (diluted 1:200). Red indicates FSHR-positive cells with immunofluorescence staining (**B**), and blue indicates the cell nucleus with DAPI staining (**C**). MERGE is the overlap combination plot of the red and blue fluorescence maps (**D**). Magnification of MERGE (**E**). Original magnification × 500.

The BGCs were morphologically intact, with clear edges, uniform in size, and polygonal or pike-shaped (e.g., Figure 1). Next, in order to verify the purification of BGCs, we detected the FSHR protein, which is the specific marker protein in BGCs. In Figure 1B–D, the red-labeled cells were the cells expressing FSHR positively; the statistical results showed that the positive rate of FSHR-expressing yak follicular GCs was greater than 95.5%, which indicated that these cells could be used for the next experiment.

### 3.2. Effect of Vitamins on Cell Viability

**Figure 2:**
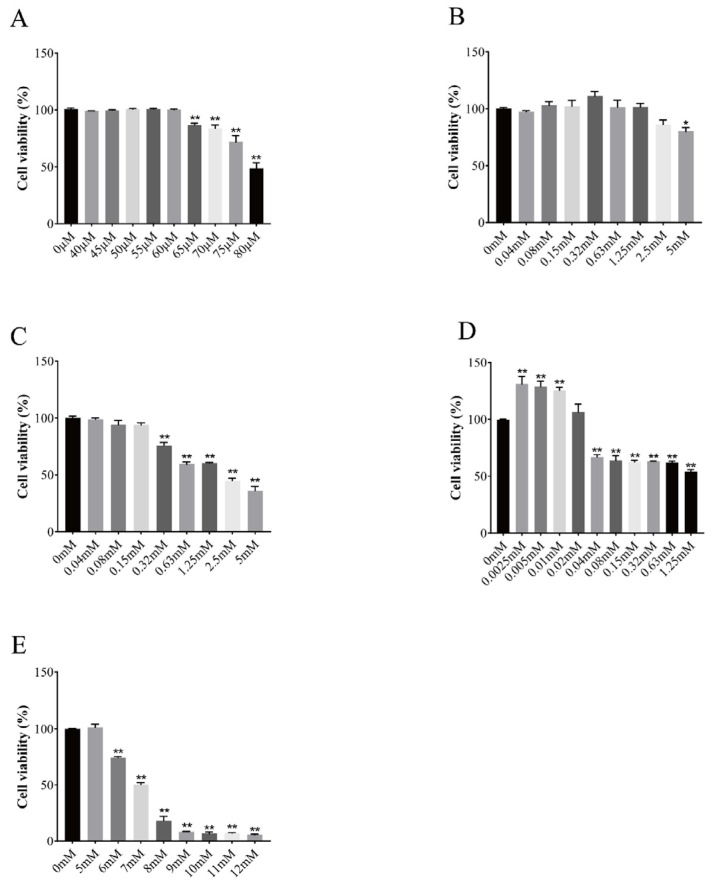
Effect of vitamins on the viability of follicular granulosa cells in yak. Changes in follicular granulosa cell activity after treatment with different concentrations of vitamin A (**A**), vitamin B12 (**B**), vitamin C (**C**), vitamin D3 (**D**), vitamin E (**E**) for 24 h. Data are presented with the means ± standard deviation (n = 6). Compared with the control group, * *p* < 0.05, ** *p* < 0.01.

The effects of different concentrations of vitamins on the activity of BGCs are shown in Figure 2. VA began to have a significant inhibitory effect on BGCs at a concentration of 65 μM; VB12 had little effect on granulosa cell proliferation and had a significant inhibitory effect at 5 mM; VC began to show a significant inhibitory effect at 0.32 mM; VD3 significantly induced the proliferation of BGCs at concentrations of 2.5 μM and 5 μM; VD3 at concentrations of 2.5 μM, 5 μM, and 10 μM significantly induced the proliferation of BGCs, but a significant inhibitory effect began to appear at 0.04 mM; VE at a concentration of 6 mM began to have a significant inhibitory effect on BGCs; and with the increase in VE concentration, BGCs activity gradually decreased.

### 3.3. Effect of Trace Elements on Cell Viability

**Figure 3:**
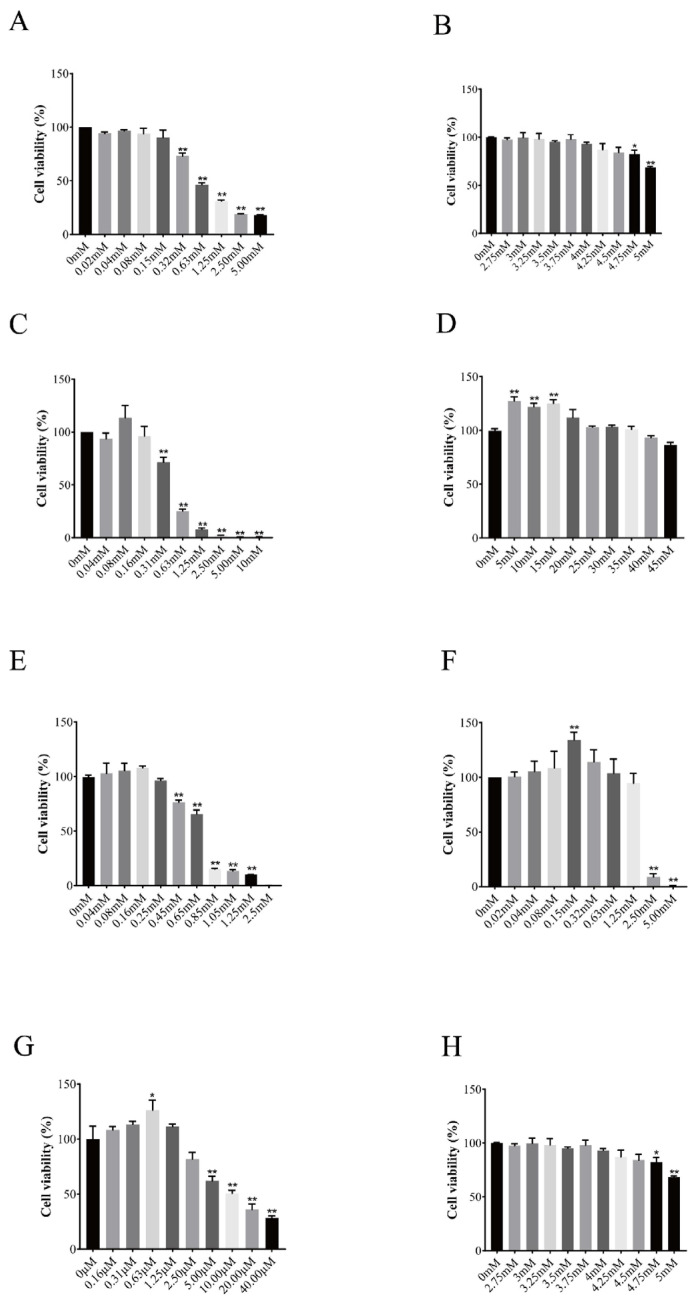
Effect of different trace elements on the activity of follicular granulosa cells in yaks. Changes in follicular granulosa cell activity after treatment with different concentrations of zinc (**A**), cobalt (**B**), nickel (**C**), strontium (**D**), copper (**E**), manganese (**F**), selenium (**G**), chromium (**H**) for 24 h. Data are presented with the means ± standard deviation (n = 6). Compared with the control group, * *p* < 0.05, ** *p* < 0.01.

The effects of different concentrations of trace elements on the proliferation of BGCs are shown in Figure 3. The results showed that Zn had an inhibitory effect on the proliferation of BGCs at 0.32 mM, and the cell activity decreased with the increase in the concentration; Co had an inhibitory effect on the proliferation of BGCs at a concentration of 0.08 mM, and the cell proliferation activity decreased with the increase in the concentration; Ni had an inducing effect on BGCs’ proliferation at a concentration of 0.08 mM but was not significant; Sr at 5 mM, 10 mM, and 15 mM significantly induced the proliferation of BGCs, but as the concentration of Sr continued to increase, there was no significant change in the proliferative activity of BGCs; Cu inhibited the proliferation of BGCs at a concentration of 0.45 mM, and the cell activity declined with the increase in the concentration; Mn significantly induced the proliferation of BGCs at 0.15 mM, and then the granulosa cell activity declined with the increase in the concentration and the cell activity declined at 2.5 mM and at 2.8 mM. Mn significantly induced the proliferation of granulocytes at a concentration of 0.15 mM, after which the granulocyte activity decreased with the increase in the concentration, and the proliferation of granulocytes was significantly inhibited at a concentration of 2.5 mM; Se significantly induced the proliferation of granulocytes at a concentration of 0.63 μM, and then the granulocyte activity decreased with the increase in the concentration; and the proliferation of granulocytes was significantly inhibited at a concentration of 5 μM of Se; and Cr significantly inhibited the proliferation of granulocytes at a concentration of 4.75 mM.

### 3.4. Effect of Vitamins on the Production of E2 and P4

**Figure 4:**
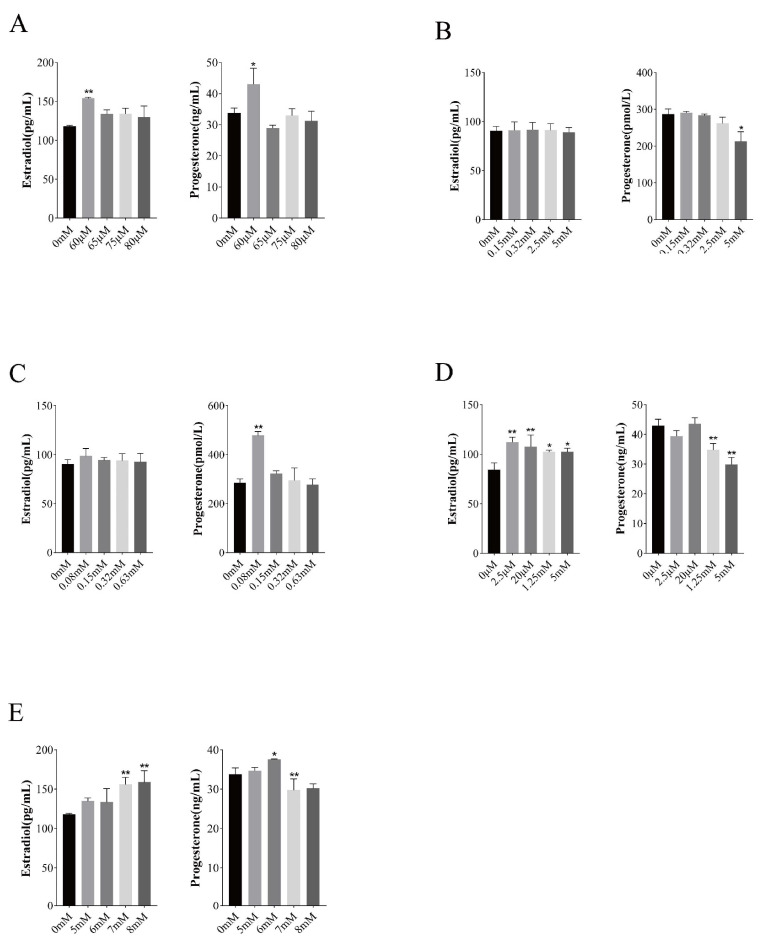
Effects of different vitamins on hormone secretion in follicular granulosa cells of yaks. Note: Changes in hormone secretion in follicular granulosa cells after treatment with different concentrations of vitamin A (**A**), vitamin B12 (**B**), vitamin C (**C**), vitamin D3 (**D**), vitamin E (**E**) for 24 h. Data are presented with the means ± standard deviation (n = 6). Compared with the control group, * *p* < 0.05, ** *p* < 0.01.

According to the effect of vitamins on the proliferative activity of GCs, suitable groups were selected, and their culture supernatants were tested for E2 and P4. The effects of different concentrations of vitamins on the synthesis of steroid hormones by BGCs are shown in Figure 4. VA significantly induced the synthesis of P4 and E2 by the BGCs at a concentration of 60 μM; VB12 significantly inhibited the synthesis of P4 and had no effect on the synthesis of E2 at a concentration of 5 mM; VC significantly induced the synthesis of P4 and had no significant effect on the synthesis of E2 at a concentration of 0.08 mM; VD3 significantly induced the synthesis of E2 and inhibited the synthesis of P4 at concentrations of 2.5 μM, 20 μM, 1.25 mM, and 5 mM, with significant inhibition of P4 synthesis at concentrations of 1.25 m and 5 mM; VE induced the synthesis of P4 at a concentration of 6 mM, significantly inhibited the synthesis of P4 at a concentration of 7 mM, and significantly induced the synthesis of E2 at concentrations of 7 mM and 8 mM.

### 3.5. Effect of Trace Elements on the Production of E2 and P4

**Figure 5:**
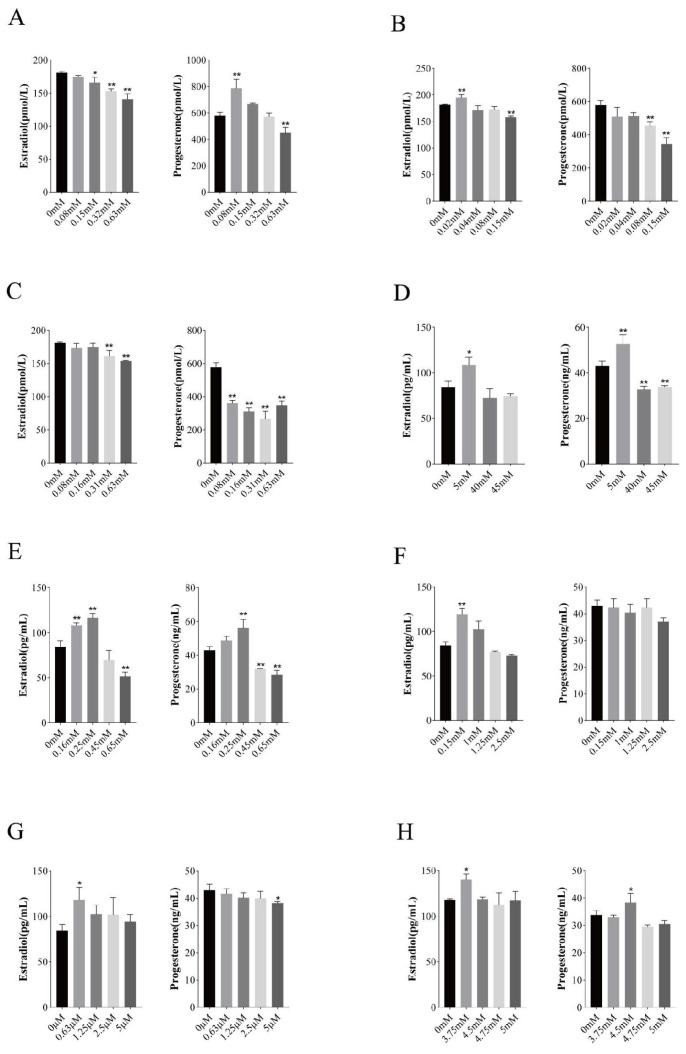
Effects of different trace elements on hormone secretion in follicular granulosa cells of yaks. Changes in hormone secretion in follicular granulosa cells after treatment with different concentrations of zinc (**A**), cobalt (**B**), nickel (**C**), strontium (**D**), copper (**E**), manganese (**F**), selenium (**G**), chromium (**H**) for 24 h. Data are presented with the means ± standard deviation (n = 6). Compared with the control group, * *p* < 0.05, ** *p* < 0.01.

According to the results of 3.3, suitable groups were selected, and their culture supernatants were tested for E2 and P4. The effects of different concentrations of trace elements on the synthesis of steroid hormones by BGCs are shown in Figure 5. Zn inhibited E2 synthesis at different concentrations, and significantly induced P4 production at a concentration of 0.08 mM; Co significantly induced E2 production at a concentration of 0.02 mM, significantly inhibited E2 production at a concentration of 0.15 mM, and Co concentrations of 0.08 mM and 0.15 mM significantly inhibited P4 production; Ni significantly inhibited E2 production at 0.31 mM and 0.63 mM, and P4 production was significantly inhibited at Ni concentrations of 0.08 mM, 0.16 mM, 0.31 mM, and 0.63 mM; Sr significantly induced the synthesis of E2 and P4 at a concentration of 5 mM, and P4 production was significantly inhibited at a concentration of 40 mM and 45 mM of Sr; Cu significantly induced E2 production at concentrations of 0.16 mM and 0.25 mM, significantly inhibited E2 production at a Cu concentration of 0.65 mM, significantly induced P4 production at a Cu concentration of 0.25 mM, and significantly inhibited P4 production at a Cu concentration of 0.45 mM and 0.65 mM; Mn significantly induced E2 production at a concentration of 0.15 mM; Se significantly induced E2 production at a concentration of 0.63 μM, and P4 generation was significantly inhibited at a concentration of 5 μM; Cr significantly induced E2 generation at a concentration of 3.75 mM, and P4 generation was significantly induced at a concentration of 4.5 mM of Cr.

### 3.6. Effect of Trace Elements on the Expression of Genes Related to Steroid Hormone Synthesis

**Figure 6:**
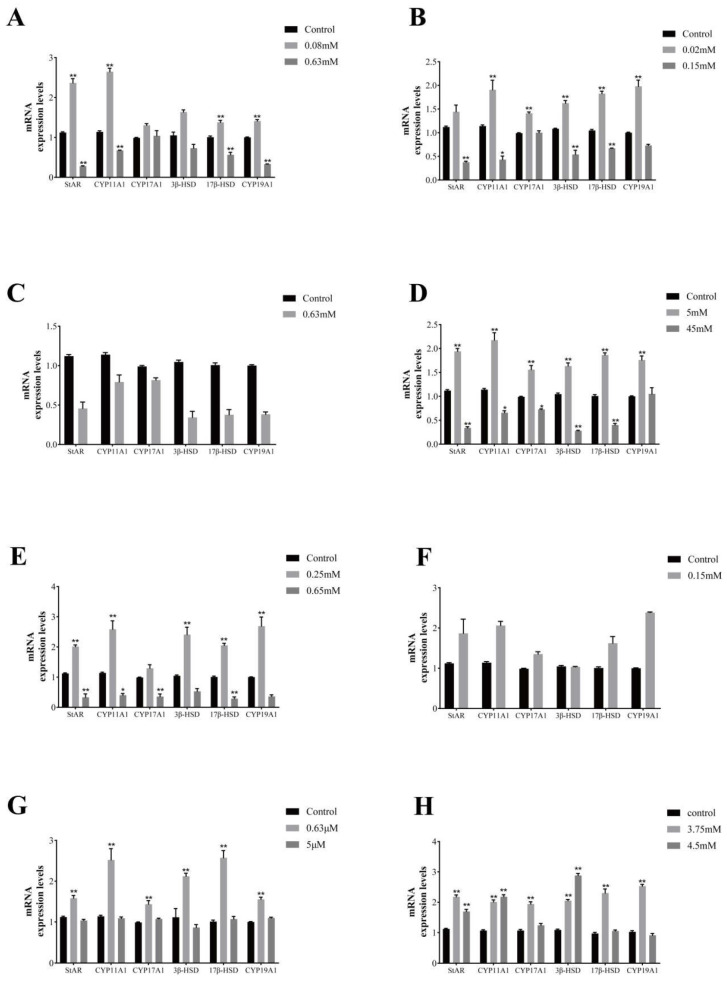
Effects of different trace elements on genes related to steroid hormone synthesis in yak follicular granulosa cells. Changes in steroid hormone synthesis genes in follicular granulosa cells after treatment with different concentrations of zinc (**A**), cobalt (**B**), nickel (**C**), strontium (**D**), copper (**E**), manganese (**F**), selenium (**G**), chromium (**H**) for 24 h. Data are presented with the means ± standard deviation (n = 6). Compared with the control group, * *p* < 0.05, ** *p* < 0.01.

We examined steroid hormone synthesis-related genes in the group with significant steroid hormone changes, and the results are shown in Figure 6. The addition of Zn (0.08 mM) to the culture medium of GCs cultured in vitro resulted in a significant increase in the expression of StAR, CYP11A1, 17β-HSD, and CYP19A1 genes, and the addition of Zn (0.63 mM) resulted in a significant decrease in the expression of StAR, CYP11A1, 17β-HSD, and CYP19A1 genes; the addition of Co (0.02 mM) resulted in a significant increase in the expression of StAR, CYP11A1, and 17β-HSD, and CYP19A1 gene expression was significantly decreased; the addition of Co (0.02 mM) resulted in a significant increase in steroid hormone synthesis gene expression, while the addition of Co (0.15 mM) resulted in a significant decrease in the expression of StAR, CYP11A1, 3β-HSD, and 17β-HSD genes; the addition of Ni (0.63 mM) resulted in a significant decrease in the expression of steroid hormone synthesis genes; the addition of Sr (5 mM) resulted in a significant increase in the expression of steroid hormone synthesizing genes, and the addition of Sr (45 mM) resulted in a significant decrease in the expression of StAR, CYP11A1, 3β-HSD, and 17β-HSD genes; the addition of Cu (0.25 mM) resulted in a significant increase in the expression of StAR, CYP11A1, 3β-HSD, 17β-HSD, and CYP19A1 genes, and the addition of Cu (0.65 mM) resulted in a significant decrease in the expression of steroid hormone synthesizing genes; the addition of Mn (0.15 mM) resulted in a decrease in the expression of StAR, CYP11A1, CYP17A1, 17β-HSD, and CYP19A1 genes; the addition of Se (0.63 μM) resulted in a significant increase in the expression of steroid hormone synthesizing genes; the addition of Cr (3.75 mM) resulted in a significant increase in the expression of steroid hormone synthesis gene expression; and the addition of Cr (4.5 mM) resulted in a significant increase in StAR and CYP11A1 gene expression.

### 3.7. Effect of Vitamins on the Expression of Genes Related to Steroid Hormone Synthesis

**Figure 7:**
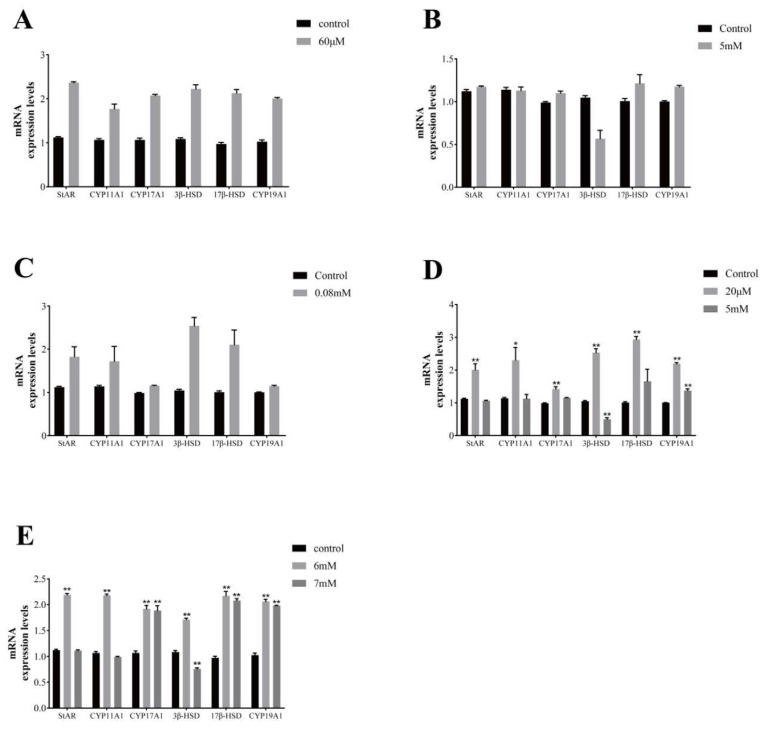
Effects of different vitamins on genes related to steroid hormone synthesis in yak follicular granulosa cells. Changes in steroid hormone synthesis genes in follicular granulosa cells after treatment with different concentrations of vitamin A (**A**), vitamin B12 (**B**), vitamin C (**C**), vitamin D3 (**D**), vitamin E (**E**) for 24 h. Data are presented with the means ± standard deviation (n = 6). Compared with the control group, * *p* < 0.05, ** *p* < 0.01.

The effects of adding vitamins to the culture solution of GCs cultured in vitro on steroid hormone synthesis genes are shown in Figure 7. Adding V_A_ (60 μM) to the culture solution of GCs cultured in vitro resulted in a significant increase in the expression of all steroid hormone synthesis genes of GCs; adding V_B12_ (5 mM) resulted in a decrease in the expression of 3β-HSD genes; adding V_C_ (0.08 mM) resulted in a significant increase in the expression of StAR, CYP11A1, 3β-HSD, and 17β-HSD gene expression; the addition of V_D3_ (20 μM) significantly increased steroid hormone synthesizing gene expression; the addition of V_D3_ (5 mM) significantly decreased 3β-HSD gene expression and significantly increased CYP19A1 gene expression; the addition of V_E_ (6 mM) significantly increased steroid hormone synthesizing gene expression; and the addition of V_E_ (7 mM) resulted in a significant increase in the expression of CYP17A1, 17β-HSD, and CYP19A1 genes and a decrease in the expression of 3β-HSD gene.

## 4. Discussion

The important link between nutrition and reproduction was clearly established many years ago. Research has shown that many trace elements are important in supporting normal reproduction in many animal species [28]. The effect of trace elements on the viability of yak follicle GCs showed that a small amount of Mn, Se, and V_D3_ supplementation can significantly promote the cell viability of yak follicle GCs. Although Sr can promote the viability of GCs, it requires a more significant concentration. So, the effect of Sr on GC viability was minor. The other trace elements did not enhance the cell viability of GCs; V_E_, V_B12_, and Cr inhibited the proliferation of GCs only when the concentration was more significant, so the effects of V_E_, VB_12_, and Cr on GCs were minor.

The main function of GCs is steroid hormone synthesization and secretion. Regarding the effect of vitamins on the synthesis of GCs, we found that, within the range of safe doses, small additions of V_A_, V_C_, and V_D3_ promoted the synthesis of steroid hormones, and V_B12_ had a lesser effect on the synthesis of steroid hormones. Although V_E_ could promote steroid hormone synthesis, it required a more significant concentration. So, it was shown that V_E_ had a lesser effect on the follicular GCs of yaks and the synthesis of steroid hormones is less influential. V_D3_ is the main form of V_D_. In recent years, more and more people have begun to realize the key role of maintaining normal physiological levels of V_D3_ in maintaining normal ovarian function and female reproductive physiology [29]. Studies have shown that V_D_ deficiency is directly related to poor fertility, and V_D3_ supplementation can improve polycystic ovary syndrome (PCOS), reduce androgen levels, and increase pregnancy rates [30,31]. Studies have shown that V_D3_ can induce the synthesis of E2 and increase the expression of steroidogenic genes in muskrat granulosa cells, which is consistent with the results of this study [26]. Similar studies in ruminants have shown that V_D_ receptors are expressed in goat ovarian GCs and that V_D3_ may play an important role in GCs proliferation by regulating cellular oxidative stress and cell cycle-related genes [32]. Some studies have shown that the addition of V_A_ can promote the synthesis of steroid hormones in porcine follicular granulosa cells. This promoting effect may be due to the regulation of ovarian steroidogenesis by RA, one of the active forms of V_A_, through enhancing granulosa cell proliferation and the MESP2/STAR/CYP11A1 pathway [33]. In cattle, V_C_ can mitigate the negative effects of heat stress on reproductive processes by regulating amino acid metabolism in granulosa cells [27]. However, there are few studies on the effect of V_C_ on the steroid hormone secretion of granulosa cells, and the specific mechanism is still unclear, which needs to be further explored.

The effect of trace elements on the synthesis of steroid hormones was also measured in this study. The results indicated that within the range of safe doses, the addition of small amounts of Co, Cu, Mn, Se, and Cr promoted the synthesis of E2 by GCs, and the addition of small amounts of Zn, Cu, and Cr significantly promoted the synthesis of P4 by GCs. Although the addition of Sr significantly promotes the synthesis of E2 and P4, the required concentration is higher, which shows that the effect of Sr on the synthesis of steroid hormones by GCs is less minor. The effect of GCs synthesizing steroid hormones was small. Zn is an essential component or activator of steroid synthase [34]. During oocyte development, Zn maintains meiotic arrest in the pre-zygotic stage of the oocyte and ensures that the egg develops to adequate maturity [35]. The results of this experiment show that Zn induces the synthesis of P4 in GCs at non-toxic concentrations. Mn is a cofactor for enzymes required for cholesterol synthesis. Steroid hormone production depends on the supply of cholesterol precursors, so Mn is important for female reproduction [36]. It has been shown that Mn promotes the proliferation of bovine GCs cultured in vitro, which is consistent with the results of this experiment [37]. The addition of Co salts to feeds can improve livestock reproduction and increase hemoglobin and other blood components. The present study’s results showed that the addition of small amounts of Co promoted the synthesis of E2, but the exact mechanism is still unclear. Our previous study showed that excess Ni induced reproductive damage in male mice. The results of the present study showed that the addition of small amounts of Ni inhibited the activity of GCs as well as the synthesis of steroid hormones [38]. It has been shown that Ni induces structural and functional changes in porcine GCs in vitro and induces inflammation and fibrosis in mouse ovaries, which is consistent with the results of the present experiment. Therefore, it can be preliminarily concluded that Ni has a negative effect on female reproduction [39,40]. Cu did not promote the proliferation of yak GCs in this study, but the results of the hormone assay showed that Cu had a significant effect on the hormone secretion function of GCs. Cu has been shown to be a potential regulator of ovarian secretory activity. In ruminants, copper addition stimulates CYP19A1 expression in ovarian granulosa cells through the AKT and WNT signaling pathways, but excess copper also disrupts steroidogenesis in ovarian granulosa cells through the FSHR/CYP19A1 pathway. This suggests that the effect of copper on BGCs is complex and needs to be further explored [23,41]. Some studies have shown that Se supplementation can also increase the gene expression levels of certain enzymes and may improve lipid metabolism, which is consistent with the results of the present experiment, and it can be tentatively hypothesized that the addition of Se has a promotional effect on the function of GCs [42]. The present experiment showed that the addition of Cr had a promotional effect on the function of yak follicular GCs, but there are fewer studies in this direction, which need to be further explored.

Steroid hormone synthesis by granulosa cells requires the coordinated expression of multiple enzymes, mainly involving two classes of enzymes: cytochrome P450 enzymes (CYP) and hydroxysteroid dehydrogenase (HSD), of which StAR is the key rate-limiting enzyme in the steroid hormone synthesis process. By examining the steroid hormone synthesis genes, we found that trace elements and vitamins may influence E2 synthesis in granulosa cells by regulating the expression of StAR and CYP19A1. In the group with increased P4 synthesis, the expression of CYP11A1 mRNA, a key rate-limiting enzyme for progesterone synthesis, significantly increased. The groups with significant decreases in hormone synthesis had varying degrees of steroid hormone synthesis genes. The results of this experiment suggest that the effects of micronutrients and vitamins on steroid hormones in granulosa cells may be regulated through genes.

## 5. Conclusions

Trace elements and vitamins are indispensable components in the reproduction process of yaks, and the present study showed that Zn (0.08 mM), Sr (5 mM), Cu (0.16 mM, 0.25 mM), Mn (0.15 mM), Se (0.63 μM), Cr (3.75 Mm, 4.5 mM), VC (0.08 mM), and VD3 (2.5 μM, 20 μM) induced GCs to synthesize steroid hormones and increased the expression of steroid hormone synthesizing genes in a small amount of the above trace elements or vitamins in yak females. VC (0.08 mM) and VD3 (2.5 μM, 20 μM) induced the synthesis of steroid hormones in GCs and increased the expression of steroid hormone synthesizing genes, so it can be preliminarily judged that the small amount of the above additions of trace elements or vitamins had a positive effect on female reproduction in yaks. This study provides an experimental basis for improving the production performance of yaks by adding trace elements.

## Figures and Tables

**Table 1:** Primer sequences for qRT-PCR used in the present study.

Gene	Accession No.	Primer Sequences (5′-3′)	Product Size	Tm (°C)
StAR	NM_174189	F: GACACGGTCATCACTCACGA	170 bp	65
		R: ACAAGGTTTCCTGCCACCTC		
CYP11A1	NM_176644	F: TTCAACCTCATCCTGACGCC	149 bp	61
		R: GTGCAAGAGGTGTGGACTGA		
CYP19A1	NM_174305	F: GGTGTCCGAAGTTGTGCCTA	148 bp	65
		R: ACCTGCAGTGGGAAATGAGG		
CYP17A1	NM_174304	F:CCATCAGAGAAGTGCTCCGAAT	80 bp	61
		R: GCCAATGCTGGAGTCAATGA		
3β-HSD	NM_174343	F:AAGACGCAACACCTCAGCAACG	197 bp	60
		R: TGGATCTGCAACACGGGCCAA		
17β-HSD	NM_001102365	F: AAGACGCAACACCTCAGCAACG	100 bp	59
		R: TGGATCTGCAACACGGGCCAA		
β-actin	NM_007393	F: GCTGTGCTATGTTGCTCTAG	117 bp	60
		R: CGCTCGTTGCCAATAGTG		

## Data Availability

The data are contained within this article.
